# Effect of Dielectric Constant on the Zeta Potential of Spherical Electric Double Layers

**DOI:** 10.3390/molecules29112484

**Published:** 2024-05-24

**Authors:** Khawla Qamhieh

**Affiliations:** Department of Physics, College of Science and Technology, Al-Quds University, Jerusalem 20287, Palestine; khawlaq@gmail.com

**Keywords:** colloidal particles, zeta potential, Monte Carlo, dielectric constant, charge inversion

## Abstract

Zeta potential refers to the electrokinetic potential present in colloidal systems, exerting significant influence on the diverse properties of nano-drug delivery systems. The impact of the dielectric constant on the zeta potential and charge inversion of highly charged colloidal particles immersed in a variety of solvents spanning from polar, such as water, to nonpolar solvents and in the presence of multivalent salts was investigated through primitive Monte Carlo (MC) model simulations. Zeta potential, ξ, is decreased with the decreasing dielectric constant of the solvent and upon further increase in the salinity and the valency of the salt. At elevated levels of salt, the colloidal particles become overcharged in all solvents. As a result, their apparent charge becomes opposite in sign to the stoichiometric charge. This reversal of charge intensifies until reaching a saturation point with further increase in salinity.

## 1. Introduction

Charged colloids dispersed in a solution are prevalent in both biological and technical systems. Examples include proteins, micelles, microemulsions, latex particles based on polystyrene, and silica particles. The physiochemical properties of these systems are controlled to a large degree by electrostatic interactions [1]. In an electrolyte solution, a charged colloidal particle is typically surrounded by small ions of opposite charge, known as counterions, which serve to balance the surface charge. The charged surface of the colloidal particle, along with the surrounding diffuse layer of ions, is commonly referred to as the electric double layer (EDL). This EDL plays a fundamental role in the stability and coagulation of dispersed systems, as described by DLVO theory [2,3]. EDL could have different geometry, such as planar [4], cylindrical [1], spherical [5], and ellipsoidal [6], depending on the charged surface geometry. Due to its widespread presence in both natural and technological realms, this phenomenon has garnered significant attention from the scientific community. It encompasses a wide range of engineering applications, including colloid stability, energy conversion, desalination, separation processes, nanofabrication, nanofluidic devices, and ion transport across membranes [7,8,9].

The electrostatic correlations among ions within the electric double layer (EDL) can give rise to a range of intriguing phenomena in systems containing multivalent ions. These may include phenomena such as charge inversion or short-range attraction between macroions of like charge. These correlations have been neglected in the DLVO theory through applying the mean field approaches [1,5,10,11,12,13].

Computer simulations and theoretical investigations have demonstrated that correlation-induced attraction or charge inversion can serve as a driving force for the aggregation of colloidal particles, even in the presence of small multivalent ions [14,15,16,17]. The universality of the implicit phenomena is understood to stem from the significant energetic contribution of Coulombic interactions to the free energy, which surpasses that of entropy and renders the system relatively insensitive to the internal structure of the counterions [16,18,19]. Martin-Molina and colleagues investigated the charge inversion of colloids in electrolyte mixtures comprising both multi- and monovalent counterions [20]. They demonstrated that incorporating short-term correlations between ions into the study of ion distribution yields markedly different results from classical treatments. In their study, charge inversion was observed in the presence of an electrolyte mixture containing both multi- and monovalent counterions. EDL properties can be affected by many factors, for example, the excluded volume of small ions [21,22] and the asymmetry in electrolyte valence [23]. Schwer and Kenndler conducted experimental research on the impact of a solvent composition’s dielectric constant on electroosmotic flow in fused-silica capillaries. They concluded that, except for acetone–water mixtures, increasing the fraction of organic solvent in water led to a decrease in zeta potential [24].

In our investigation, we utilized Monte Carlo (MC) simulations to explore the properties of spherical electric double layers (EDLs) surrounding highly charged colloidal particles immersed in various solvents, ranging from polar solvents like water to nonpolar ones. Our aim was to examine the influence of dielectric constant on the zeta potential and charge inversion of colloidal particles in the presence of multivalent salts, including trivalent and pentavalent ions. The study of spherical EDLs is particularly pertinent due to their significance in dispersions of globular proteins, micelles, polymer beads, dendrimers, and other closely spherical organic or inorganic macroions [23]. The structure of the article is outlined as follows: Section 2 provides details on the model and parameters settings used for the numerical calculations. Section 3 presents the results and discussion derived from the simulations conducted. The conclusions are outlined in the fourth section.

## 2. Results and Discussion

### 2.1. Systems without Salts

**Figure 1:**
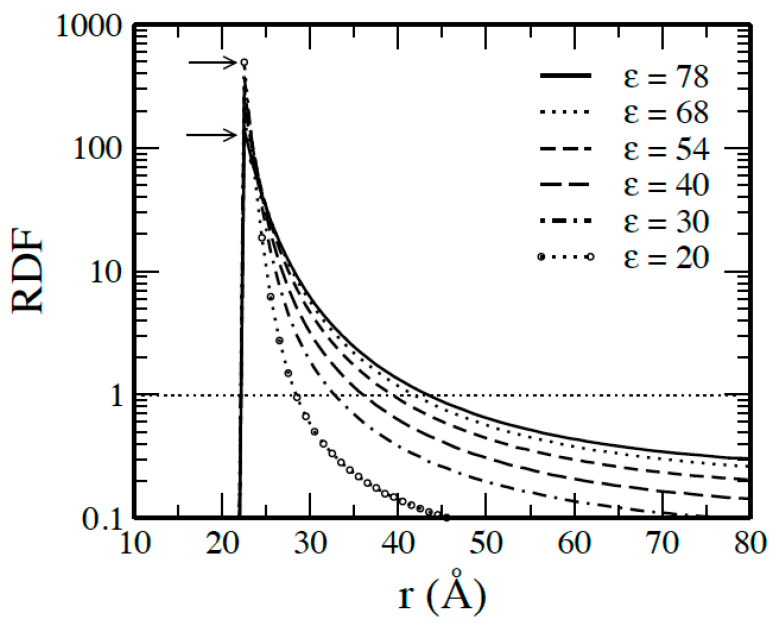
Macroion counterion radial distribution functions for 60:1 system at indicated values of the dielectric constants *ε*. The arrows point to the values at *r* = *R_M_* + *R_I_* = 22 Å. The dotted horizontal line represented at *g_MI_* corresponds to uncorrelated particles in the systems.

Figure 1 illustrates the macroion counterion radial distribution functions (RDFs) describing the relative densities of counterions at a distance *r* from the macroion for the 60:1 system in different solvents, with different dielectric constants as in Table 1. The horizontal dotted line exemplifies systems of uncorrelated particles. The figure shows a maxima at the hard sphere contact separation *r* = *R*_M_ + *R*_I_ = 22 Å for the macroion–counterion RDFs curves demonstrating the accumulation of the counterions close to the macroion. At *ε* = 20, where Γ=3, the value of the maximum is 505 and decreases monotonically as the dielectric constant of the solvent is increased and gives a value of 120 at *ε* = 78 where Γ=0.8, meaning the maximum is decreased as the electrostatic intensity is decreased.

The accumulated running charge $Z_{acc}$(*r*), within a distance *r* from the center of the macroion, is an important quantity that follows directly from the macroion–ion radial distribution function $g_{MI}$ according to the following:

(1) $$Z_{acc}\left(r\right)=Z_{M}+\int_{r}^{\infty }\sum [Z_{i}\rho_{i}g_{Mi}\left(r\prime \right)]4\pi r\prime^{2}dr\prime$$

where $\rho_{i}$ is the uniform number density of the corresponding species. The accumulated running charge can be used to calculate the mean electrostatic potential at a distance *r* from the center of the macroion:

(2) $$\varphi \left(r\right)=\int_{r}^{\infty }dr\prime E(r^{\prime })=\frac{e}{4\pi \varepsilon \prime }\int_{r}^{\infty }dr^{\prime }\frac{Z_{acc}\left(r^{\prime }\right)}{r{`}^{2}}$$

The upper cutoff in Equation (2) is taken to be 60 Å since the typical accumulated charge rapidly decays to zero [25]. The surface potential $\varphi_{s}$ is the electric potential at the surface of the macroion, i.e., $\varphi_{s}=\varphi (R_{M})$, while the zeta potential ξ is identified with the diffuse potential at the slipping plane or hydrodynamic shear surrounding a particle surface and located one ionic diameter away from the macroion, i.e., $\xi =\varphi (R_{M}+2R_{I})$. 

**Figure 2:**
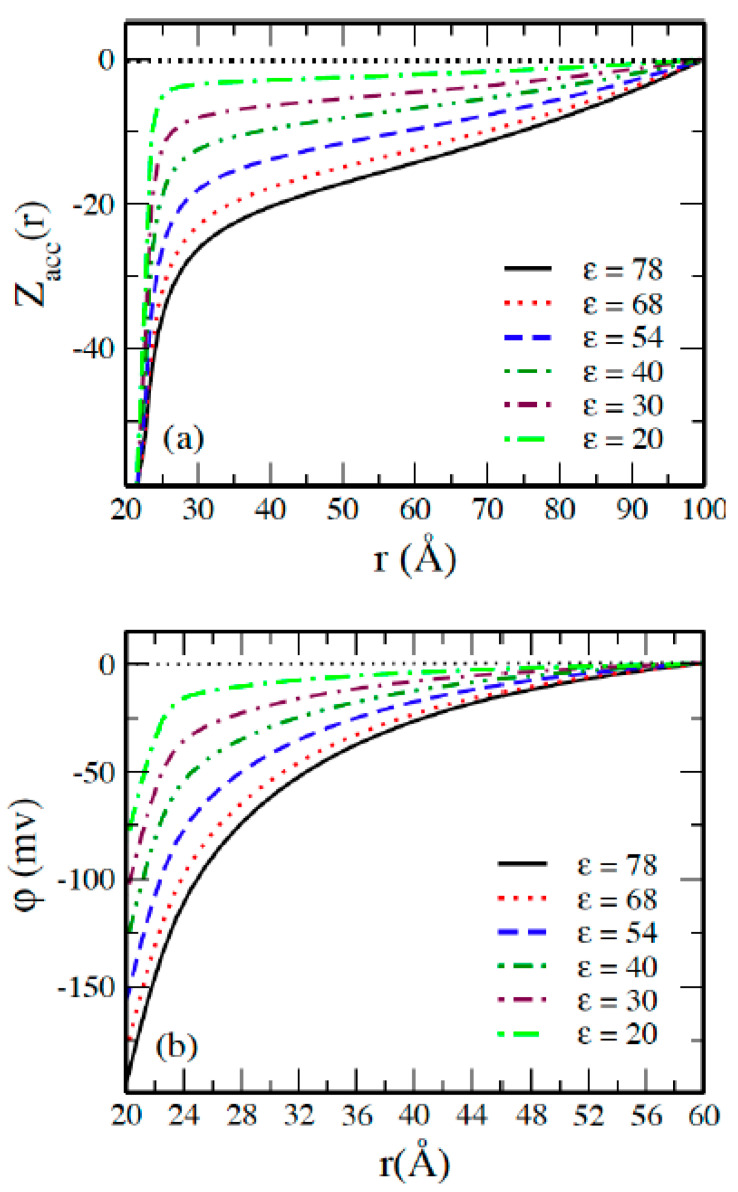
(**a**) Accumulated running charge *Z_acc_*(*r*) and (**b**) mean electrostatic potential *φ*(*r*) of the macroion EDL as a function of distance *r* from the macroion center for 60:1 system in solvents with the indicated dielectric constants *ε*.

**Figure 3:**
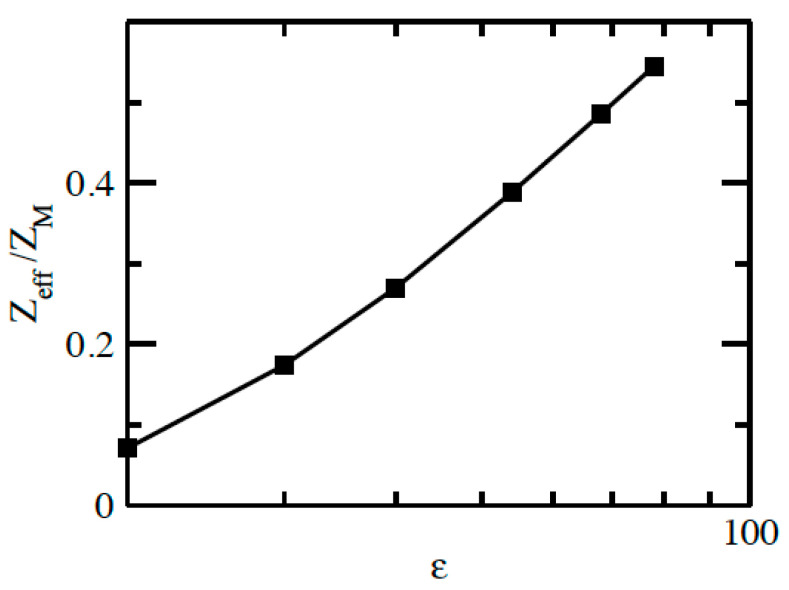
Effective macroion charge for 60:1 system in different solvents as a function of the dielectric constant *ε*.

The accumulated running charge curves are presented in Figure 2a, which shows that the total charge decays monotonically and reaches zero at the cell boundary in all systems, and the decay rate increases by changing the solvent from water as a polar solvent to a nonpolar one, thereby decreasing the dielectric constant. This can be explained by the escalation in the counterion–counterion coupling parameter Γ as the dielectric constant of the solvent is decreased, as shown in Table 1. The negative value of the mean electrostatic potential $\varphi \left(r\right)$ of the macroion EDL, shown in Figure 2b, is rapidly decreased when the polarity of the solvent is decreased as a consequence of decreasing the effective charge of the macroion $Z_{M}^{eff}$ by decreasing the dielectric constant, as illustrated in Figure 3, where $Z_{M}^{eff}=$ *Z*_acc_(*R* + 2*R*_I_). The value of $Z_{M}^{eff}$ starts from about 0.54 $Z_{M}$ at *ε* = 78.4 with water solvent and then decreases to reach 0.07 $Z_{M}$ at the lowest dielectric constant *ε* = 20.

**Figure 4:**
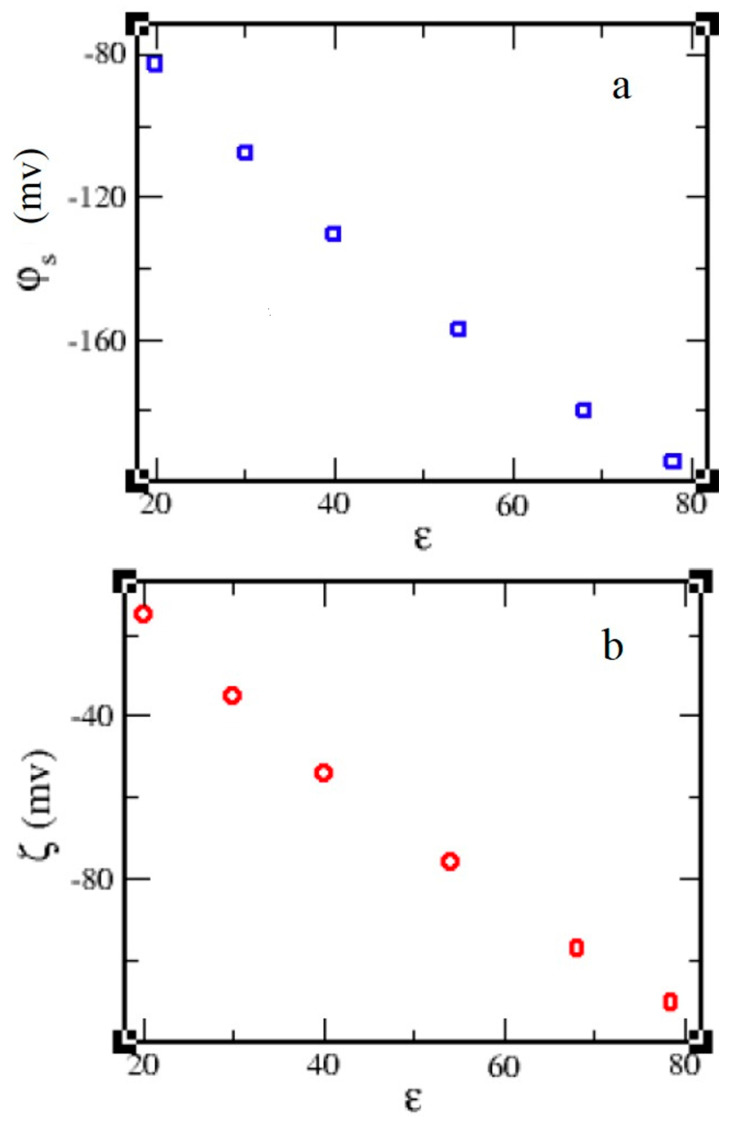
(**a**) Surface potential *φ_s_* and (**b**) zeta potential *ξ* of EDL as a function of *ε* for 60:1 system in different solvents without salt.

Figure 4 shows that the negative values of the surface $\varphi_{s}$ and the ξ potentials are linearly decreased by decreasing the dielectric constant. Our finding for ξ potential is in good agreement with the experimental result concluded by Schower et al. [24], where they found that ξ potential is decreased as the fraction of organic solvent in water is increased, i.e., by decreasing the polarity of the solvent, the dielectric constant of the solvent is consequently decreased.

The decrease in *ξ* potential is a natural consequence of the increased accumulation of counterions around the macroion in solvents with smaller dielectric constants compared to those with greater ones. Since the zeta potential represents the electrostatic potential at the shear plane within the electric double layer *EDL*, a decrease in the dielectric constant of the solvent results in a stronger electric field, a screening to the Coulomb force, and a less effective charge at the macroion surface. Consequently, the zeta potential decreases as the dielectric constant of the solvent decreases.

**Figure 5:**
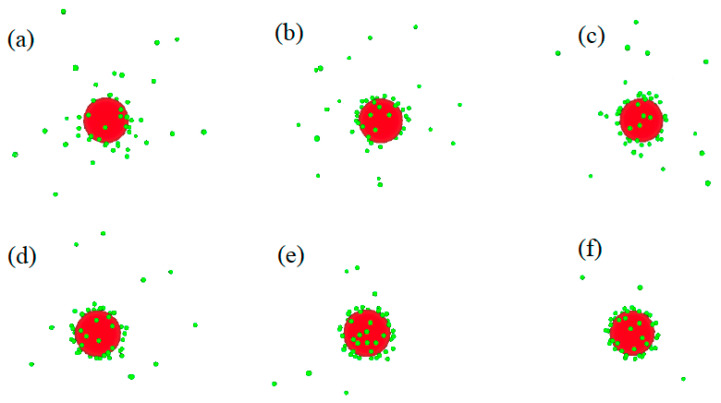
Counterions’ (small green balls) distribution around the macroion (large red ball) for 60:1 system in solvents with different dielectric constants: (**a**) *ε* = 78, (**b**) *ε* = 68, (**c**) *ε* = 54, (**d**) *ε* = 40, (**e**) *ε* = 30, and (**f**) *ε* = 20.

Figure 5 illustrates snapshots of the distribution of counterions around the macroion for the 60:1 system in solvents with varying dielectric constants. In Figure 5a, the smallest accumulation of counterions around the macroion is observed in water with a dielectric constant of *ε* = 78. As the dielectric constant decreases to *ε* = 68 (Figure 5b), *ε* = 54 (Figure 5c), *ε* = 40 (Figure 5d), *ε* = 30 (Figure 5e), and *ε* = 20 (Figure 5f), the accumulation of counterions around the macroion increases, reaching its maximum in solvents with a dielectric constant of *ε* = 20.

The greater accumulation of the counterions around the macroion leads to a decrease in the effective charge of the macroion $Z_{M}^{eff}$ on which ξ potential depends.

### 2.2. Systems with Salt

**Figure 6:**
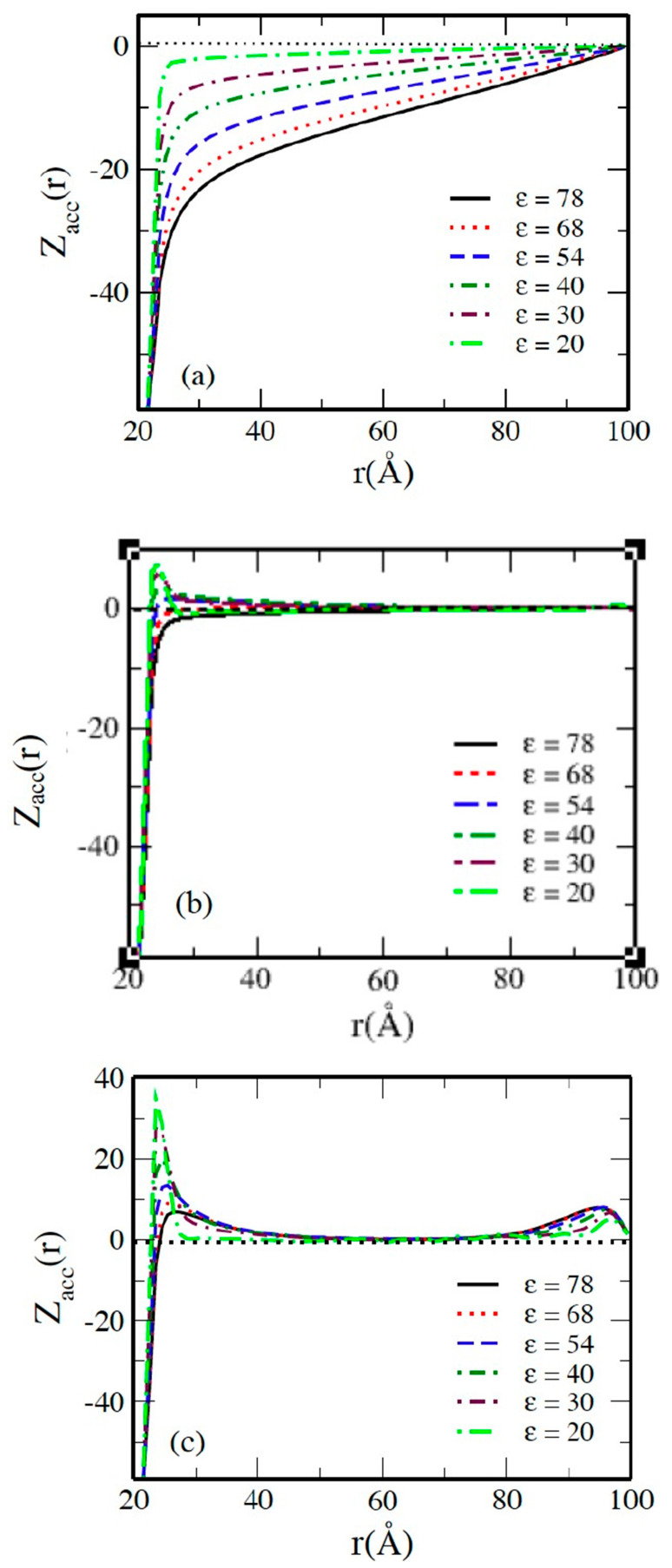
Accumulated running charge *Z_acc_*(*r*) as a function of the distance *r* from the macroion center in solvents with the indicated dielectric constants *ε* and salt concentration of (**a**) *β* = 0.15, (**b**) *β* = 1, and (**c**) *β* = 6.25.

Figure 6 shows the accumulated running charge $Z_{acc}$(*r*) as a function of the distance *r* from the center of the macroion for the 60:1 system in solvents with different dielectric constants at different 3:1 salt concentrations. There is a distinct qualitative difference between the systems as salt concentration is increased. At low salt concentration *β* = 0.15, the total charge decays monotonically and reaches zero only at the cell boundary in all systems, and the decay rate increases with the decreasing polarity of the solvent, meaning the dielectric constant is decreased, as shown in Figure 6a. At higher salt concentrations, the charge shows a sharp drop to zero within a very narrow region *r* < 48 Å. At *β* = 1, $Z_{acc}$(*r*), as shown in Figure 6b, remains close to zero up to the cell boundary in solvents with a relatively high dielectric constant, like water. In solvents with lower dielectric constants, the charge changes its sign, reaches a maximum, and then decays slowly, similar to what occurs for the charges in systems with higher salt concentration, e.g., *β* = 6.25, as shown in Figure 6c. The figure shows a maximum of charge equal to ≈+37 at *ε* = 20 and then a monotonic decrease as the dielectric constant of the solvent is increased, giving a value of ≈+7 at *ε* = 78. Close to the cell boundary, the figure shows a buildup in the cation charge because of the repulsion between the charge-inverted macroions and the cations.

**Figure 7:**
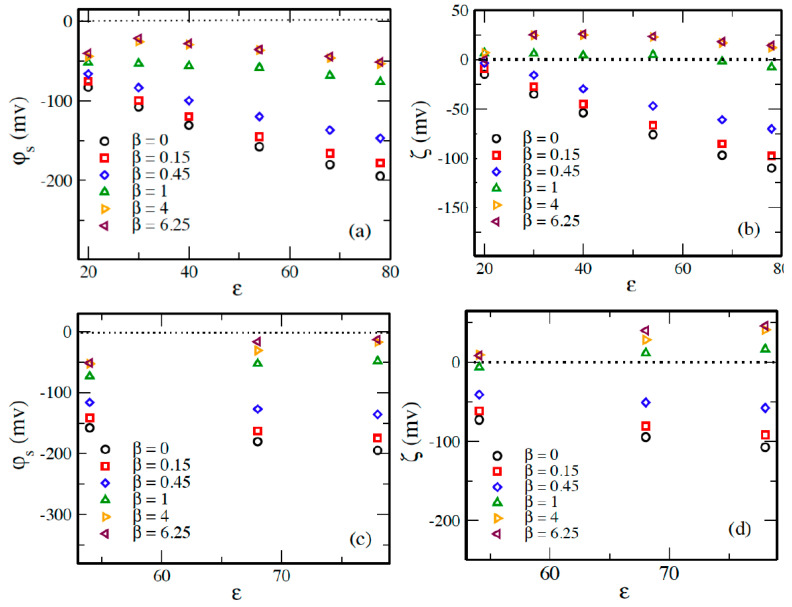
(**a**,**c**) Surface electrostatic potential *φ_s_*, and (**b**,**d**) zeta potential *ξ* of EDL as a function of *ε* for 60:1 system in different solvents at different ((**a**,**b**) 3:1 and (**c**,**d**) 5:1) salt concentrations.

Figure 7 illustrates surface electrostatic potential $\varphi_{s}$ and zeta potential ξ of EDL as a function of *ε* for 60:1 system in different solvents and at different 3:1 and 5:1 salt concentrations. The decrease in the absolute values of these potentials is almost linear when *ε* is changed from 20 to 78, which is a consequence of the decrease in the intensity of the electrostatic correlations between counterions on the surface of the macroion. 

The values of $\varphi_{s}$ and ξ potentials at *β* = 4 are very close to those at *β* = 6.25, and the potentials at *β* = 0, *β* = 0.15, and *β* = 0.45 are much less negative than those at *β* < 1. At *β* = 1, the ξ potential is close to zero, corresponding to neutralized macroions, while its values are inverted to positive with salt concentrations β > 1, where charge inversion occurs. The decrease in the potential due to the changing salt concentration is largest when *ε* = 78, while it is smallest when *ε* = 20. The decrease is larger in the systems with 5:1 salt, where ξ is changed from ≈−110 mv to ≈+45 mv at *ε* = 78, while it is changed to ≈+13 mv in the systems with 3:1 salt, meaning that the charge inversion is larger with 5:1 salt than it is with 3:1 salt, as the coupling parameters are larger in systems with 5:1 salt. 

Our findings hold significant promise for practical applications, especially in the realm of drug delivery systems. Through manipulation of the salt concentration and dielectric constant of the nanoparticle suspension, we can effectively control zeta potential of the nanoparticles. This zeta potential is a critical determinant of the efficacy of nanomedicine. It plays a pivotal role in various processes such as the binding of nanoparticles to cell membranes, drug release behavior in vitro, and the stability of nanoparticles suspensions. Variations in zeta potential have the capacity to regulate their binding to tissue and guide nanoparticles (NPs) to specific cellular compartments both in vitro and in vivo [26,27]. In vitro drug release behavior can be managed by controlling the solubility, pH, and zeta potential of the material [28]. Zeta potential serves as a marker for the stability of NP suspensions [29]. Generally, *ξ* potential values exceeding 30 mV indicate good stability for low molecular weight surfactants and pure electric stabilization, while values surpassing 60 mV signify excellent stability. Zeta potential values around 20 mV offer only short-term stability, whereas those ranging from −5 mV to +5 mV suggest rapid aggregation. For higher or larger molecular weight stabilizers, which primarily function through steric stabilization, *ξ* values of only 20 mV or even lower can provide adequate stabilization [30].

**Figure 8:**
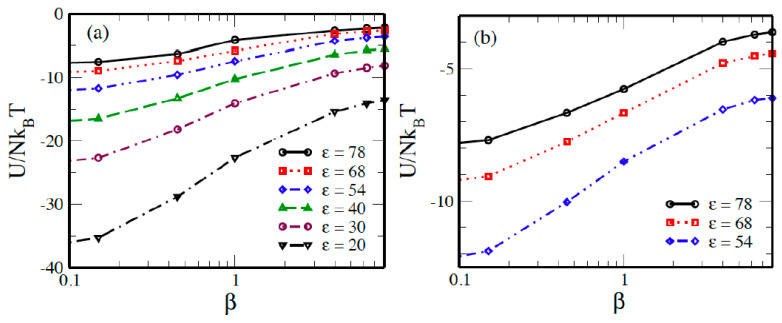
Reduced electrostatic energy as a function of (**a**) 3:1, and (**b**) 5:1 salt concentration *β* for a 60:1 system in various solvents with different dielectric constants *ε*.

In Figure 8, we illustrate the reduced electrostatic energy of the solution *U*/*N*$k_{B}$*T* for the 60:1 system, once with a 3:1 salt concentration (Figure 8a) and another with a 5:1 salt concentration (Figure 8b), in various solvents with differing dielectric constants, where *N* represents the total number of ionic species. Figure 8 demonstrates that the total potential energy is consistently negative across all systems due to the strong attraction between the macroion and counterions. This energy decreases monotonically with the salt content, reflecting the simple consequence of an increasing overall number of particles, and is more negative with a 5:1 salt concentration compared to a 3:1 salt concentration at specific salt concentrations. All curves exhibit similar shapes with varying curvatures, with an observed inflection point at approximately *β* = 1 on each curve. The reduction in magnitude is less pronounced with the 5:1 salt concentration than with the 3:1 salt concentration.

## 3. Model and Method

### 3.1. Model

The macroion is represented as a hard sphere of radius *R*_M_ = 20 Å and has a charge of valence *Z*_M_ = −60 that is impeded at the center of a spherical cell of radius 100 Å, including asymmetric electrolytes described within the scope of the primitive model, whereas the solvent enters the model by its dielectric constant ε while maintaining a constant temperature of T=298 K. These parameters maintain the systems at a macroion number density of $\rho_{M}=2.5\times 10^{-7}\;{Å}^{-3}$ corresponding to a macroion volume fraction of $\emptyset_{M}=0.008$. They also provide a highly charged macroion with a surface charge density of $\sigma =0.19\;C/m^{2}$ to guarantee a strong electrostatic interaction by which the charge inversion of the macroion occurs. The dielectric constants ε, and the corresponding Bjerrum lengths $l_{b}$ for the solvents used in our study are shown in Table 1. The electrolyte also contains small ions of radius *R*_I_ = 2 Å and charge valence *Z*_I_ = +1, representing the counterions, while the solvent enters by its dielectric constant. The added salt consists of small ions of radius 2 Å, monovalent anions of *Z*_a_ = −1, and cations of different valences: trivalent ones of *Z*_c_ = +3 and pentavalent cations of *Z*_c_ = +5. The amount of added salt is characterized by the ratio of the total added cation charge to the total macroion charge, $\beta =Z_{c}\;\rho_{c}/{(Z}_{M}\;\rho_{M})$, where *ρ_c_* is the number density of the corresponding species. In the study, many values of *β* between 0.15 to 8 were used. 

Simulation, while often costly and time-consuming, becomes more manageable with primitive models. These models simplify molecular interactions, leading to faster computations compared to more detailed approaches. This efficiency facilitates the study of larger systems over longer time scales. Despite their simplicity, primitive models are adept at capturing essential aspects of system behavior, revealing fundamental physical principles’ governing properties. The streamlined nature of primitive models enables systematic exploration of parameter space, facilitating the study of various factors influencing system behavior. While they may not capture quantitative details accurately, primitive models provide qualitative insights and predict trends in system behavior. Although primitive models offer valuable insights, they also come with limitations. They often oversimplify molecular interactions, neglecting crucial details such as atomic configurations and bond orientations. Consequently, inaccuracies may arise, particularly in describing subtle effects or interactions. Additionally, primitive models may assume a constant ionic strength, which may not reflect real-world variations. Interaction potentials used in these models might not encompass all relevant forces present in the system, and solvation effects may be inadequately accounted for. Overall, while primitive models may lack the detail and accuracy of more advanced models, they offer valuable insights into complex systems and serve as essential tools for theoretical and computational studies.

In our model, the electrostatic interaction between the particles is pairwise additive, and for pair *ij*, it is given by the following:

(3) $$U_{ij}\left(r\right)=\left\{\begin{array}{r} \infty ,\;\;\;\;r_{ij}<(R_{i}+R_{j})\\ \frac{Z_{i}Z_{j}e^{2}}{4\pi \varepsilon_{0}\varepsilon }\frac{1}{r_{ij}},\;\;\;\;\;r_{ij}\geq (R_{i}+R_{j}) \end{array}\right.$$

where *i* and *j* denote either the polyion or counterion. The intensity of the electrostatic correlations between counterions on the surface can be characterized by the counterion-counterion coupling parameter $\Gamma =Z_{I}^{2}l_{b}/a_{Z}$ and can be increased by lowering the solvent dielectric constant; $l_{b}=e^{2}/4\pi \varepsilon_{0}\varepsilon k_{B}T$ is the Bjerrum length, where *e* is the elementary charge, $k_{B}$ is the Boltzman constant, and *T* is the temperature in kelvin; and $a_{Z}={\left[Z_{I}/\left(\sigma /e\right)\right]}^{1/2}$ is the average distance between two neighboring counterions on a surface that has σ surface charge density, and it is known that the correlation-induced attraction appears at $\Gamma >\Gamma^{*}$ ≈ 2 [13,31]. The dielectric constants, the Bjerrum lengths for the solvents used in our study, and the corresponding Coulomb coupling parameters (Γ) between monovalent, trivalent, and pentavalent counterions on the charged surface in all solvents are shown in Table 1, which illustrates the increments in the counterion–counterion coupling parameter Γ, meaning an increment in the intensity of the electrostatic correlations between counterions on the surface of the macroion, as the valence of the counterion is increased and as the dielectric constant of the solvent $\varepsilon_{r}$ is decreased. 

### 3.2. Method and Simulation Settings

In our study, the Monte Carlo (MC) simulation method was applied and employed the canonical *NVT* ensemble with periodic boundary conditions and Ewald summation for treating the electrostatic interactions. We performed 10^6^ MC moves per particles in the production run, while for systems at *β* = 6.25, we performed up to 10^5^ moves. The macroion was fixed at the center of a spherical cell, while the small ions were initially placed randomly inside it. The simulation software MOLSIM v3.1, developed by Per Linse and colleagues, was utilized in this study. Additional information regarding the simulation methodology can be found in reference [32].

## 4. Conclusions

We performed a numerical investigation on electrolyte solutions with significant asymmetry, comprising highly charged colloidal particles with a surface charge density of $\sigma =0.19\;C/m^{2}$, in different solvents with dielectric constants of 78, 68, 54, 40, 30, and 20, once without salt and another in different amounts of multivalent salt. Decreasing the polarity of the solvent without salt, i.e., deceasing *ε*, gradually lowers the effective charge, the surface potential $\varphi_{s}$, and the zeta potential of the colloidal particles. ξ is changed from ≈−110 mv at *ε* = 78 to ≈−15 mv at *ε* = 20. The addition of minute quantities of multivalent salt yields a similar effect in all solvents. By changing the salt concentration, the decrease in ξ potential is largest when *ε* = 78, while it is smallest when *ε* = 20. The decrease is larger in the systems with 5:1 salt, where ξ is changed from ≈−110 mv to ≈+45 mv at *ε* = 78, while it is changed to ≈+13 mv in the systems with 3:1 salt, meaning that the charge inversion is larger with 5:1 salt than it is with 3:1 salt, as the coupling parameters are larger in systems with 5:1 salt. At *β* = 1, the ξ potential is close to zero, corresponding to neutralized macroions, while its values invert to positive with salt concentrations β > 1 where charge inversion occurs.

Currently, colloidal nano-carriers are undergoing rapid growth owing to their considerable potential in resolving persistent challenges like poor drug solubility and bioavailability. Moreover, they exhibit limitless possibilities in drug targeting. For instance, the binding of nanoparticles to cell membranes appears to be significantly influenced by the surface charge of the particles, which is reflected in their zeta potential, ξ [26,27]. The variation in ξ potential of the particles holds the potential to govern their binding to tissue and direct nanoparticles (NPs) to specific cellular compartments both in vitro and in vivo. In vitro drug release behavior can be managed by controlling the solubility, pH, and zeta potential of the material [28].

Zeta potential serves as an indicator of the stability of nanoparticles suspensions [29]. Typically, for low-molecular-weight surfactants and pure electric stabilization, absolute ξ potential values exceeding 30 mV indicate good stability, while values surpassing 60 mV signify excellent stability. Zeta potential values around 20 mV provide only short-term stability, while those ranging from −5 mV to +5 mV indicate rapid aggregation [30].

Our findings are of immense significance, as zeta potential is one of the key properties that could profoundly impact the effectiveness of future nanomedicine and the design of drug delivery systems.

## Figures and Tables

**Table 1:** Dielectric constants ε, Bjerrum lengths for the solvents used $l_{B}$, and the corresponding counterion–counterion coupling parameter (Γ) between the monovalent, trivalent, and pentavalent counterions on the charged surface in all solvents used in our study.

The Solvent	Water	(75% Water, 25% Ethanol)	Methyl Alcohol	Glycerin	Methanol	Ethanol
Dielectric constant (ε)	78	68	54	40	30	20
$l_{B}$ (Å)	7.1	8.2	10.4	14.0	18.7	28.0
$\Gamma_{1}$	0.8	0.9	1.1	1.5	2.0	3.0
$\Gamma_{3}$	4.1	4.7	5.7	7.8	10.4	15.6
$\Gamma_{5}$	8.9	10.1	12.3	16.8	22.4	33.5

## Data Availability

The original contributions presented in the study are included in the article, further inquiries can be directed to the corresponding author.
